# Inhibitior of Bcl6 by FX1 protects DSS induced colitis mice through anti-inflammatory effects

**DOI:** 10.3389/fimmu.2025.1558845

**Published:** 2025-05-09

**Authors:** Ruixian Liu, Zhe Zhang, Chuan Zhou, Binbin Wang, Muhan Zhang, Yaxing Sun, Yao Yao, Yanru Zhang, Yijia He, Junzhi Yu, Yimeng Xia, Yan Liu, Shiyang Ning, Baisui Feng

**Affiliations:** Department of Gastroenterology, the Second Affiliated Hospital of Zhengzhou University, Zhengzhou, China

**Keywords:** BCL6, IBD, pro-inflammatory cytokines, macrophage, tight junction proteins (TJPs)

## Abstract

**Introduction:**

Inflammatory bowel disease (IBD) is a complex immune-mediated condition, and biologics are the most commonly used drugs for its treatment. However, there are still cases of ineffective treatment. B-cell lymphoma 6 (Bcl6), a transcriptional suppressor, is known to have regulatory effects on multiple immune-associated cell subsets. FX1, a novel specific BCL6 Bric-à-brac (BTB) inhibitor, has shown positive effects in many disease models, but its effects and mechanisms in IBD control remain unclear.

**Methods:**

We observed colon length and DAI score of colitis mice after treatment. HE staining section was used to evaluate colonic injury, while the expression of colonic pro-inflammatory cytokines by RT-qPCR. And differences in immune cell subsets between the two groups was analyzed by flow cytometry. Additionally, IHC and RT-qPCR were employed to evaluate the expression of colonic tight junction proteins. Furthermore, RAW264.7 cells and co-cultured Caco2 cells were detected by ELISA and RT-qPCR.

**Results:**

In the treat group, colitis symptoms in mice were significantly improved, and there was a decrease in proportion of macrophages and protection of intestinal mucosal integrity-indicating anti-inflammatory effects of FX1. In cell experiments, we found that FX1 decreased secretion of pro-inflammatory factors by macrophages and increased expression of tight junction proteins in Caco2 cells after co-culture.

**Discussion:**

The experimental findings demonstrate the inhibitory effect of FX1 on inflammation in murine colitis model as well as its potential mechanism. BCL6 is a potential target for treating IBD.

## 1 Introduction

Crohn’s disease and ulcerative colitis, belonging to inflammatory bowel disease (IBD), are complex immunologically mediated diseases that arise due to a dysregulated immune response to commensal flora in a genetically susceptible host (1).

The prevalence of IBD is steadily increasing over time due to its chronic nature and rising incidence, as well as the global trend towards aging. This increase in prevalence is not only related to morbidity but also reflects the overall growth of IBD cases (2). Since 1990, the highest reported prevalence values have been observed in Europe and North America. Although still lower in East Asian compared with Western countries, incidence has been on the rise in newly industrialized countries in Africa, Asia, and South America, including Brazil. This suggests that changing environmental factors play an important part (3). IBD not only has a physical impact on patients’ lives, but also leads to higher levels of anxiety, depression, and sleep disturbances, further reducing their quality of life. Therefore, it is crucial to explore new mechanistic targets in IBD in order to facilitate the development of clinically effective drugs (4).

Bcl6 (B-cell lymphoma 6) is a transcriptional repressor that plays a critical role in regulating germinal centers (GC) and T cell differentiation, as well as the production and maturation of germinal center B cells (5). Bcl6 encodes a BTB domain at its amino terminus, which is essential for the transcriptional inhibition of several proteins, including SMRT and NCOR (6). Bcl6 not only prevents premature activation and differentiation of GC B cells (7), but also plays a key role in the development and differentiation of T cells, especially Tfh cells (8). Bcl6 is a key transcription factor that regulates germinal center B cell differentiation and germinal center response, and is important for the development of memory type CD8^+^ T cells and follicle-killing CD8^+^ T cells (7). In addition, Upregulation of Bcl6 is associated with the expression of the chemokine receptor CXCR5, which allows Tfh cells to migrate to the T-B boundary and GCs region, where they interact with B cells of the specific antigen (9). It follows that Bcl6 plays a role in regulating Tfh development and is critical for Tfh cell differentiation and function (9).

Existing studies have demonstrated that Tfh cells can be regulated through the modulation of the Bcl-6/Blimp-1 signaling pathway, thereby influencing the incidence of IBD (10). Furthermore, Wei et al. discovered that activation of the Bcl6/Syk/BLNK signaling pathway in mice with DSS-induced colitis can regulate the balance of memory B cell subsets, prevent excessive accumulation of inflammatory cells, and mitigate immune damage (11). As an important factor in inflammatory and autoimmune diseases (12, 13), Bcl6 may provide a new idea for the treatment of IBD. Compared with gene editing, the intervention method of small molecule inhibitors is easier to achieve clinical conversion application (14). The Bcl6 inhibitor, FX1, exhibits a 10-fold greater potency than endogenous corepressors and is the most efficient among Bcl6 small molecule inhibitors. FX1 disrupts Bcl6 recruitment of corepressors to its endogenous target genes and induces derepression of Bcl6 target genes and transcriptional program. Hence, FX1 can broadly disrupt the BCL6 transcriptional program in cells (15, 16). Previous studies have shown that in mice with sepsis, FX1 can reduce the condition by inhibiting Bcl6 in macrophages (17). However, the role of BCL6 in macrophages within the context of IBD remains unclear.

Based on the previous research, Bcl6 has been mentioned as a potential therapeutic target for patients with IBD (18). However, the therapeutic role of FX1 in mice with colitis remains unclear. The objective of this study was to investigate the therapeutic effects of FX1 on colitis both *in vivo* and *in vitro*, as well as to elucidate its possible mechanisms. Our findings indicate that FX1 exhibits anti-inflammatory effects in DSS-induced colitis mice and LPS-induced cell inflammation experiments.

## 2 Materials and methods

### 2.1 Ethics statement

For all experiments, we used 8–10-week-old wild-type (WT) C57BL/6 male mice. The WT mice were obtained from Liaoning Changsheng Biotechnology Co., LTD (Benxi, China) and were housed at the Medical science research institute of Henan province. The housing conditions included a temperature of 22–23°C and a 12-hour light/dark cycle, with ad libitum access to food and water. The experiments were approved by the Ethics Committee of the Medical science research institute of Henan province(Ethical code: 2024-yyy-030).

### 2.2 Dextran sulfate sodium-induced colitis models

Sixteen C57BL/6J mice were fed for 1 week in the Experimental Animal Center of Zhengzhou University and randomly divided into two groups as colitis mouse model treatment group and control group. A mouse model of acute colitis was established by free drinking of 2.5% dextran sulfate sodium (DSS) solution (MP Biomedicals, USA) for 6 days. Mice in the treatment group received a daily intraperitoneal injection of 25mg/kg FX1 solution(Selleck, S8591), while the control group was injected with a DMSO carrier. Clinical progression of colonic inflammation in mice was assessed using a disease activity index (DAI) score based on body weight, stool, and occult blood (19) (**Supplementary Table S1**). The DAI is calculated as follows: DAI = (weight loss score + stool character score + hematochezia score)/3 (20), and the DAI score ranges from 0 to 4, with higher scores indicating more severe disease activity. After modeling and treatment, the psychophysiological state of the mice was monitored daily through assessing signs of lethargy, fatigue, inactivity, crouching behavior, and reduced activity levels. Additionally, food intake, weight fluctuations, and stool characteristics were systematically recorded, and fecal occult blood was tested from day 3. On the seventh day, anesthetize mice via intraperitoneal injection of room temperature 20 mg/ml Avertin (0.45 mg/g body weight). Avertin administration timing is set at 5 minutes per mouse. The mice were subsequently assessed for toe reflexes to confirm that they were fully anesthetized. The mice were euthanized by cervical dislocation. Colons, spleens, mesenteric lymph nodes, and peripheral blood samples were collected. Changes in colon length were measured.

### 2.3 HE

After the colon was rinsed with PBS at 4°C, it was fixed in 4% paraformaldehyde and then embedded in paraffin for sectioning and histological evaluation (**Supplementary Table S2**). The sections were sliced to a thickness of 4µm, dried, and subsequently treated with xylene and gradient alcohol. Following this, the sections underwent staining using the hematoxylin-eosin routine (Servicebio, Wuhan, China) and were observed under a microscope with tissue panoramic scanner (GCell, Guangzhou, China) for image capture (21).

### 2.4 IHC

The paraffin-embedded slices underwent incubation at 100°C for 30 min. Following this, the samples were sequentially treated with 100% xylene, 95% xylene, 95% ethanol, 95% ethanol, and 100% ethanol, each for 5 min. Antigen repair of the slices was performed using citrate buffer and EDTA. The tissue was incubated with a 3% hydrogen peroxide solution for 25 minutes to block endogenous peroxidase. It was then uniformly covered with 3% BSA and left at room temperature for 30 minutes. After incubation with the primary antibody at 4°C overnight (ZO1: abcam, AB221547,1:500/Occludin: abcam, AB216327,1:200), the secondary antibody was incubated at room temperature for 50 minutes. DAB color development was performed, and hematoxylin was re-dyed for 3 minutes.

The staining results were semi-quantitatively evaluated based on staining intensity and the percentage of positive staining cells. Staining intensity was assessed using a four-point scale: 0 for negative, 1 for weak, 2 for moderate, and 3 for strong. The percentage of positive cells was categorized into four grades: 0 indicating <5%, 1 representing 6%-25%, 2 representing 26% to 50%, 3 representing 51% to75%, and 4 representing >75%. Each section was analyzed in three independent amplification fields to determine the average score product (22).

### 2.5 Flow cytometry

After 6 days of treatment, samples were collected from the colon, mesenteric lymph nodes, peripheral blood, and spleen on the day 7. The colon was cultured in a digestive medium, while the spleen and peripheral blood underwent treatment with red blood cell lysate before being passed through a 70-micron filter. The lymph nodes were milled in PBS and also passed through a 70-micron filter. Cells were then incubated with antibody cocktails to identify different immune cells populations. The first regimen of antibodies combination is CD45-Percp, CD3-BV510, CD4-FITC, CD127-APC, CD25-PE, CCR4-PECy7, CD183-BV650, CCR6-PE Dazzle TM594, CD161-Alexa Fluor 700, CD8-BV711, TCR-BV421(Biolegend). The second regimen is CD45-Percp, CD11b-FITC, F4/80-PEcy7, MHCII-BV605, CD163-PE DAZZLE 594, CD3-BV510, CD49b-APC, Gr1-PE, GP38-BV421, CD90.2-BV785, CD11C-BV711, CD19-BV650 (Biolegend). Samples were detected on a BD FACSAria™ III (BD Biosciences) and data was analyzed by FlowJo software version 10.8.1.

### 2.6 Single-cell analysis and t-SNE analysis

The website (https://singlecell.broadinstitute.org/single_cell) provides a dataset on single-celled CD data set (PREDICT 2021 paper: CD) for single cell analysis. The t-SNE dimensionality reduction analysis of streaming data is conducted using Flowjo software.

### 2.7 Cell lines

The Caco2 cell line, purchased from QuiCell Biological Technology Co., LTD (Shanghai, China), was maintained in Dulbecco’s modified Eagle’s medium (DMEM) with 20% fetal bovine serum (FBS). The RAW 264.7 cell line was purchased from Servicebio (Wuhan, China), and maintained in DMEM with 10% FBS (heat inactivated).

Both companies offer 5×10^5^ cells at the time of purchase. All cells were cultured in an incubator at 37°C with a 5% CO2 atmosphere.

The 12-well plates were incubated overnight with RAW 264.7 cells at a concentration of 5 × 10^5^/ml. The cells were then serum-starved in DMEM for 3 hours, followed by replacement with DMEM containing 10% FBS and incubation with either 50 μM FX1 or DMSO for 4 hours. Subsequently, the cells were stimulated with LPS (1 μg/mL) for 24 hours. The supernatant was collected for ELISA, and the cells were frozen in TRIzol at -80°C for RT-qPCR.

### 2.8 Cell viability

Caco2 cells were seeded in 96-well plates at a density of 1.0 × 10^4^ cells/well for 24 h, and then treated with different concentrations of FX1 for 4 h. Cell counting kit-8 (CCK-8) kit (Servicebio, Wuhan, China) was used to measure the viability of treated cells as described by the manufacturer. The absorbance at 450 nm of solutions was measured by a multimode plate reader.

### 2.9 Cell co-culture

RAW 264.7 cells were laid on 12-well plates, the experimental group or control group were treated with FX1 or DMSO, and the supernatant was collected after 24h stimulation with LPS. The 12-well plates were incubated overnight with Caco2 cells at a concentration of 2 × 10^5^/ml, and were added the supernatant from RAW 264.7 when the density of Caco2 reached 70%. After incubation for 12h, Caco2 cells were collected to extract RNA for RT-qPCR assay.

### 2.10 RT-qPCR

Total RNA extraction from tissues and cells were carried out with the TRIzol reagent. The PrimeScript™ II 1st Strand cDNA Synthesis Kit (Cat #RR6210A, Takara, Japan) was used to reverse transcribe RNA. Quantitative real-time polymerase chain reaction (RT-qPCR) test was conducted by TB Green Premix Ex Taq II (Cat #RR820A, Takara, Japan) using real-time PCR Detection System (QuantStudio 5 Real-Time PCR Software, Thermo Fisher Scientific, USA).

The comparative Ct M method formula 2−ΔΔCt was used to determine relative gene expressions of TNFα, IL-1β and IL6, based on endogenous control gene GAPDH. The sequences of the primers used are shown in **Table 1**.

**Table 1:** Primers for RT-qPCR.

Gene	Primer	Sequence (5’-3’)
IL6(M)	Forward	CTGCAAGAGACTTCCATCCAG
	Reverse	AGTGGTATAGACAGGTCTGTTGG
IL-1β(M)	Forward	TTCAGGCAGGCAGTATCACTC
	Reverse	GAAGGTCCACGGGAAAGACAC
TNFα(M)	Forward	CTGAACTTCGGGGTGATCGG
	Reverse	GGCTTGTCACTCGAATTTTGAGA
Zo1(M)	Forward	GACCTTGATTTGCATGACGA
	Reverse	AGGACCGTGTAATGGCAGAC
ZO1(H)	Forward	AACTGGGCTCTTGGCTTGCTATTC
	Reverse	TCCAGAAGTCAGCACGGTCTCC
Occludin(M)	Forward	ACACTTGCTTGGGACAGAGG
	Reverse	AAGGAAGCGATGAAGCAGAA
OCCLUDIN(H)	Forward	AACTTCGCCTGTGGATGACTTCAG
	Reverse	GACCTTCCTGCTCTTCCCTTTGC
Gapdh(M)	Forward	TTCACCACCATGGAGAAGGCCG
	Reverse	GGCATGGACTGTGGTCATGA
GAPDH(H)	Forward	ACCCACTCCTCCACCTTTGACG
	Reverse	TCTCTTCCTCTTGTGCTCTTG

### 2.11 ELISA

IL-1β、TNFα or IL6 protein levels were quantified using IL-1β mouse (88-7013A-88, Thermo Fisher Scientific, USA), TNFα mouse uncoated ELISA (88-7324-88, Thermo Fisher Scientific, USA) or mouse IL6 uncoated ELISA (88-7064-88, Thermo Fisher Scientific, USA), according to the manufacturer’s instructions. Data (pg/mL) were normalized to the IL-1β standard, TNF-α standard or IL6 standard provided by the kit.

### 2.12 Statistical analysis

Statistical analysis was performed using GraphPad Prism 8.0. For the intergroup analysis of animal experiments, first evaluate whether normal distribution is satisfied. If it is, Student’s t test is used, otherwise Mann-Whitney test is used. Univariate analysis of variance was used for intergroup analysis of cell experiments. P <0.05 was considered as significant difference.

## 3 Results

### 3.1 Bcl6 inhibitor alleviates inflammatory symptoms in mice with DSS induced acute colitis.

**Figure 1:**
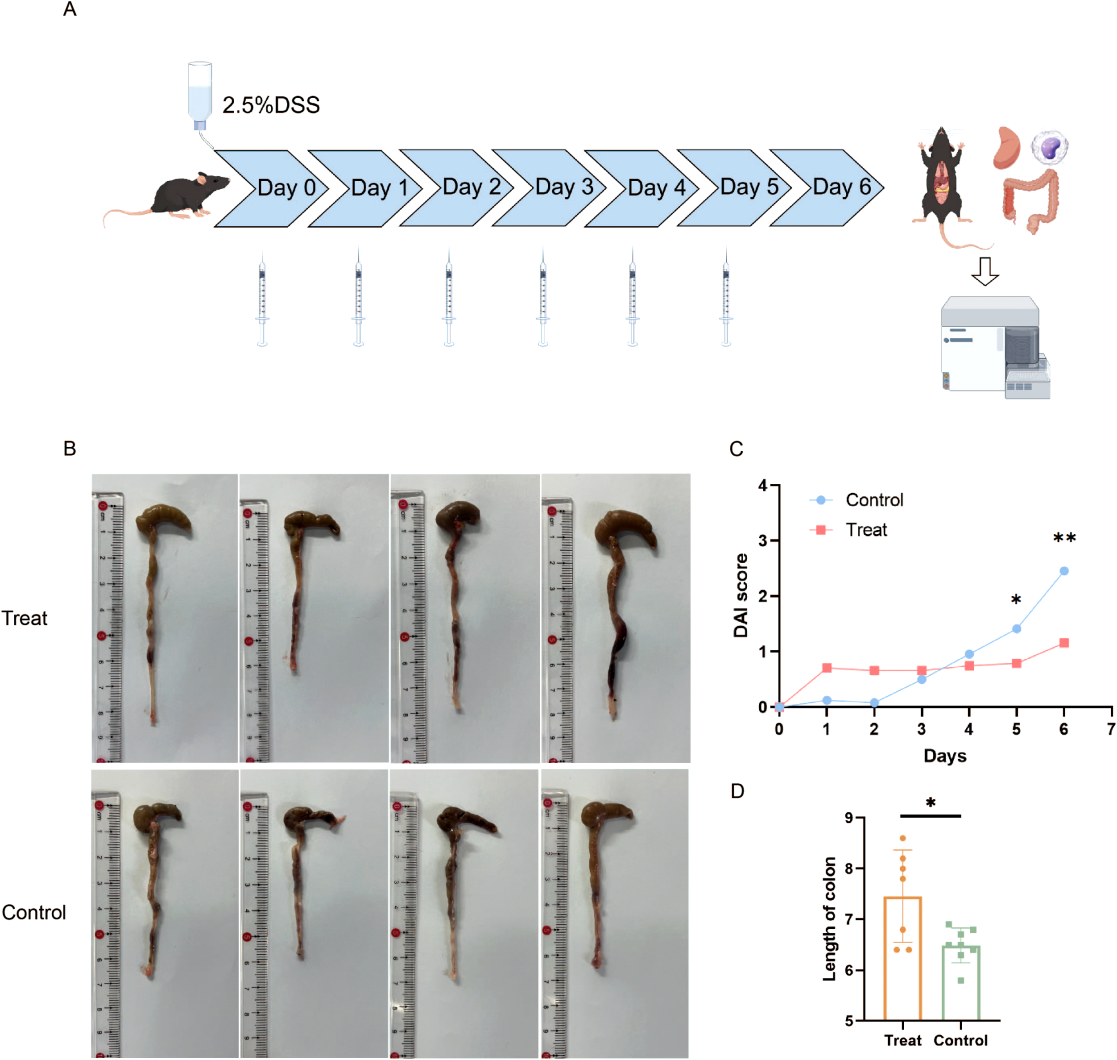
Effects of FX1 on mice with DSS induced colitis. **(A)** Flow chart of animal experiment (By Figdraw). **(B)** Comparison of colon length in mice. **(C)** Histogram of DAI score in mice. **(D)** Colon length histogram of mice. *p < 0.05; **p < 0.01.

We conducted animal experiments utilizing BCL6 inhibitors. Sixteen male C57BL/6J mice were randomly divided into a treatment group and a control group. All of them were given a 2.5% DSS solution for one week, and the vector DMSO or FX1 (25 mg/kg) was injected intraperitoneally every day (**Figure 1A**). To investigate the effect of FX1 on DSS colitis, we conducted daily monitoring of body weight and fecal characteristics in both groups of mice, while we began testing for fecal occult blood on day 3. After the molding, the colorectum was removed for comparison, and other parts were removed for follow-up experiments. Occult blood was recorded daily during the modeling process, and the results showed that FX1 treatment resulted in a significant reduction in fecal blood. Compared with the control group, the DAI score of the DSS model FX1 treatment group was significantly reduced, and the colon length was increased (**Figures 1B-D**). Our findings suggest that FX1 treatment may be effective in alleviating the symptoms of colitis.

### 3.2 Bcl6 inhibitor alleviated intestinal inflammation and reduced pro-inflammatory cytokines expression in mice with colitis.

**Figure 2:**
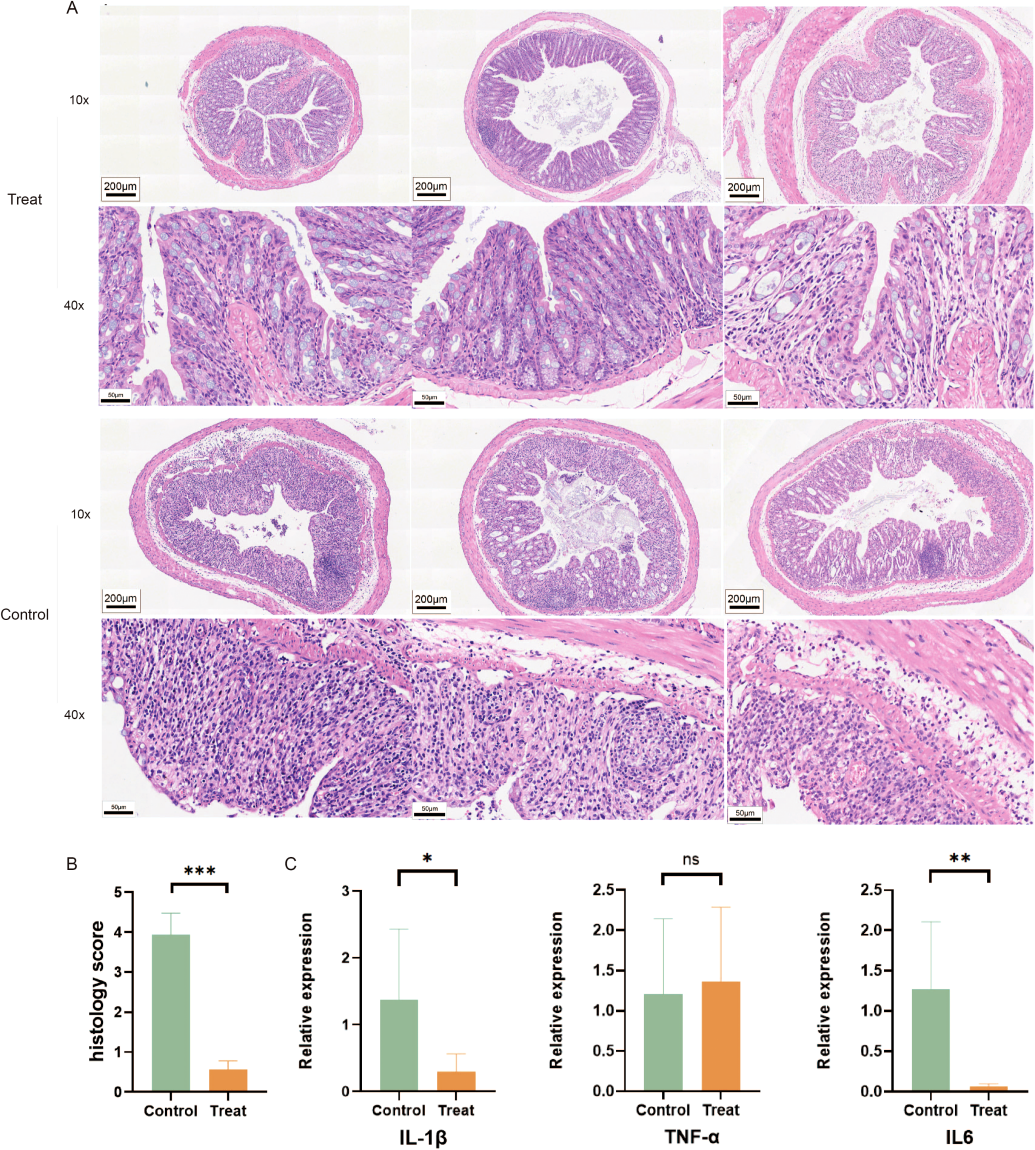
FX1 inhibits intestinal inflammation in mice induced by DSS. **(A)** HE sections of colon were obtained from treated and control groups(The left, middle and right 3 images in each row were randomly selected from 3 representative mouse colon sections from either the Treat or Control groups). **(B)** Pathological injury score histogram of mice (n=8). **(C)** RT-qPCR histograms of inflammatory cytokines in mouse colon tissue (n=5). The results were represented as mean ± SEM. *p < 0.05; **p < 0.01; ***p < 0.001; ns, no significance.

After seven days of feeding with 2.5% DSS, tissue samples measuring 0.5 cm in length were taken from the distal end of each mouse’s colon, embedded in paraffin, sectioned, and subjected to immunohistochemical and histopathological examination. HE staining revealed that, in comparison to the FX1 treatment group, mice in the control group exhibited varying degrees of colonic ulcers, necrosis and detachment of intestinal mucosa cells, as well as swelling and destruction of crypts. Goblet cells were notably absent, and inflammatory cell infiltration of varying degrees was observed in the mucosa and submucosa (**Figures 2A, B**). To investigate the impact of FX1 on the inflammatory state of colorectal in mice, RT-qPCR was conducted using RNA extracted from each mouse’s colorectal tissue (**Figure 2C**). The results showed that the levels of pro-inflammatory cytokines IL6 and IL-1β were significantly lower in the treatment group compared to the control group, while there was no significant difference in TNFα.

Our findings suggest that Bcl6 inhibitor treatment can effectively alleviate inflammatory pathological changes in the colon.

### 3.3 Bcl6 inhibitor mitigates intestinal barrier damage during acute colitis in mice with DSS

**Figure 3:**
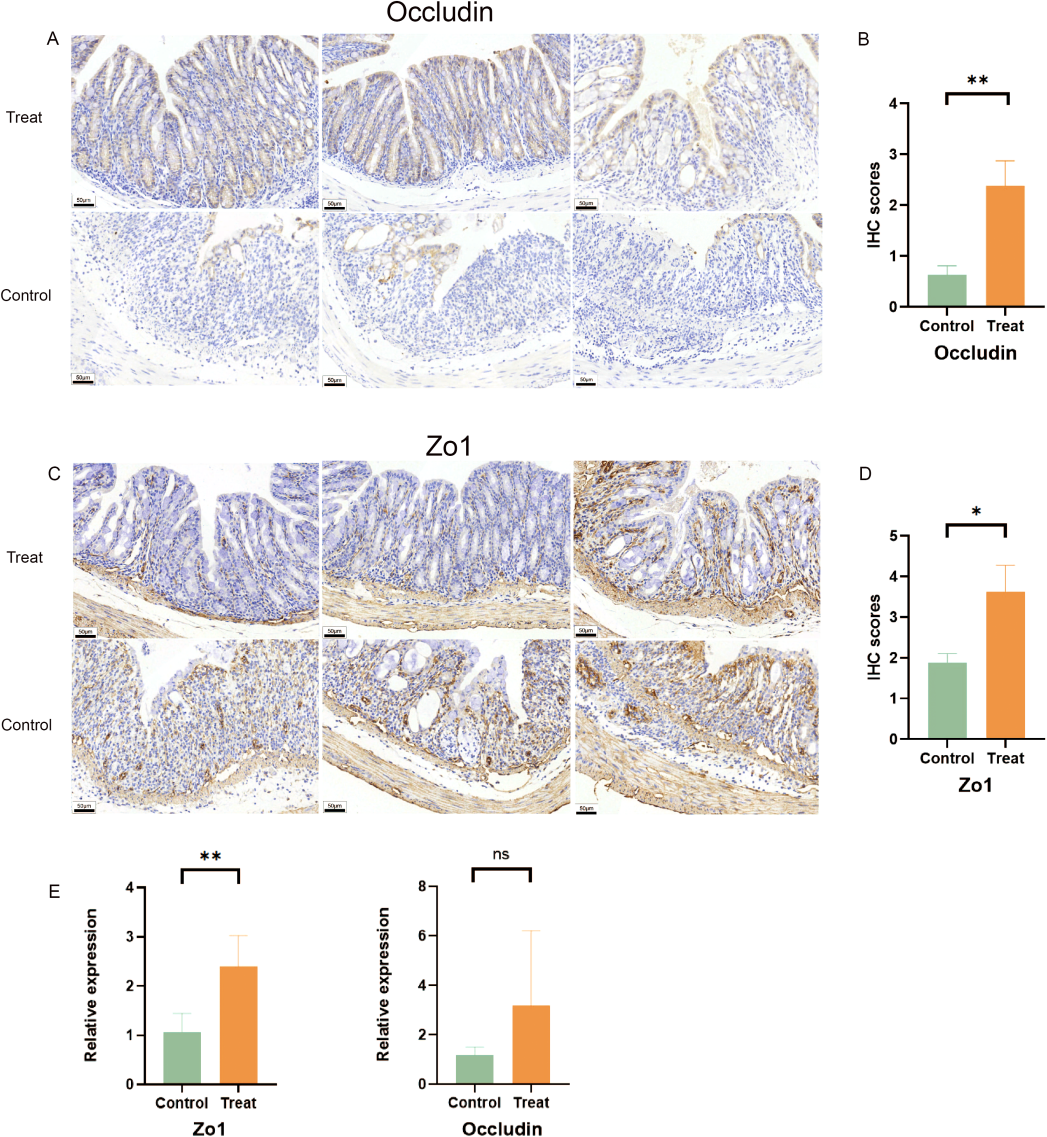
FX1 mitigates intestinal barrier damage during acute colitis in mice with DSS. **(A)** IHC sections of colon were obtained from treated and control groups(Occludin). **(B)** IHC score histogram of Occludin (n=8, the results were represented as mean ± SD). **(C)** IHC sections of colon were obtained from treated and control groups(Zo1). **(D)** IHC score histogram of Zo1 (n=8, the results were represented as mean ± SD). **(E)** RT-qPCR histogram of tight junction proteins in mouse colon tissue (n=5). The results were represented as mean ± SD. *p < 0.05; **p < 0.01; ns, no significance.

ZO-1 and occludin are well-established transmembrane proteins that play a critical role in maintaining the integrity of tight junction barrier function (23). Consequently, they are commonly utilized to assess intestinal epithelial barrier integrity (24). The IHC results revealed a higher expression of Zo1 and Occludin in the treatment group (**Figures 3A-D**). Specifically, Zo1 and Occludin were predominantly localized between the colonic tissue surface and crypt structure, exhibiting a neat and continuous distribution pattern. In contrast, the control group exhibited disordered and discontinuous distribution of these two tight-linking proteins, with complete loss observed in some regions.

Furthermore, FX1 treatment led to an increase in the expression of intestinal tight junction protein Zo1 in mice compared to those injected with DMSO only, which is consistent with previous IHC results (**Figure 3E**). Although there was no significant difference observed in Occludin, FX1 also demonstrated a trend towards improving their expression levels in the treatment group. These findings suggest that FX1 inhibits DSS-induced acute colitis and reduces inflammatory cytokine production, thereby alleviating damage to the intestinal barrier.

### 3.4 Bcl6 inhibitor affects chemotaxis of macrophages during acute colitis in mice

**Figure 4:**
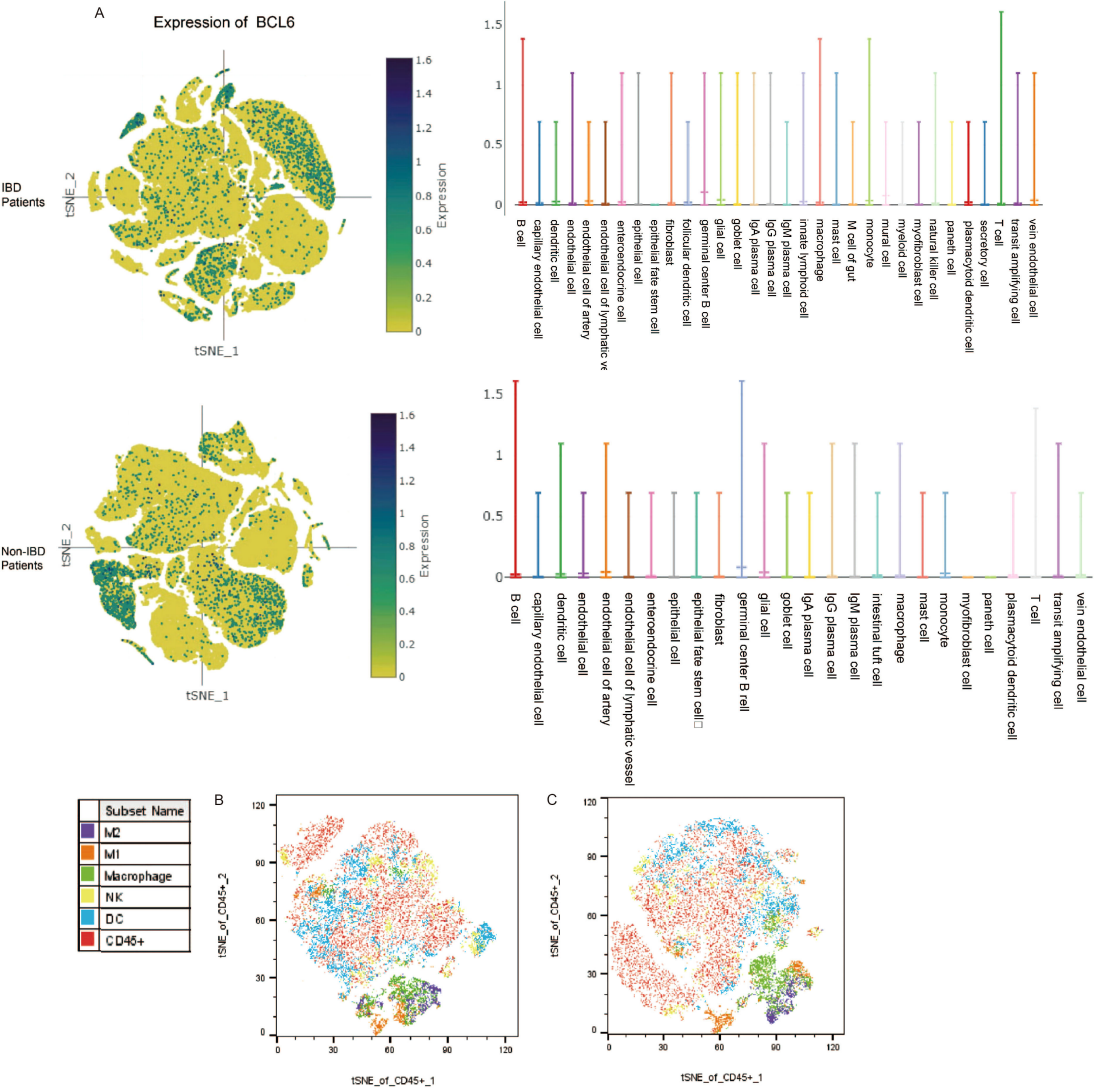
Database and flow cytometry analysis. **(A)** Expression of BCL6 in intestinal subsets of IBD patients and non-IBD patients. **(B)** t-SNE analysis of intestinal immune cell subpopulations by flow cytometry of mice in the treated group. **(C)** t-SNE analysis of intestinal immune cell subpopulations by flow cytometry of mice in the control group.

We obtained biopsy single-cell analysis data from the Single Cell Portal for patients with IBD and functional gastrointestinal disorders (FGID). We analyzed the expression levels of BCL6 across different cell types in both patient groups (**Figure 4A**, **Supplementary Figure S1**). The expression level of BCL6 was found to be highest in B cells, while remaining relatively high in T cells and mononuclear macrophages. Furthermore, we utilized FlowJo to perform t-SNE dimensionality reduction visualization on experimental data from intestinal tissues (**Figures 4B, C**). Notably, compared to the Treat group, the proportion of intestinal macrophages in the Control group was significantly higher, which may be influenced by the BCL6 inhibitor.

Given the effective reduction of colonic inflammatory response in mice treated by FX1, we conducted an evaluation of the effects of FX1 on activation clustering and infiltration of immune cells in DSS-fed mice. After seven days of 2.5% DSS feeding, samples from the spleen, colorectal tissue, and mesenteric lymph nodes were collected and analyzed using flow cytometry.

**Figure 5:**
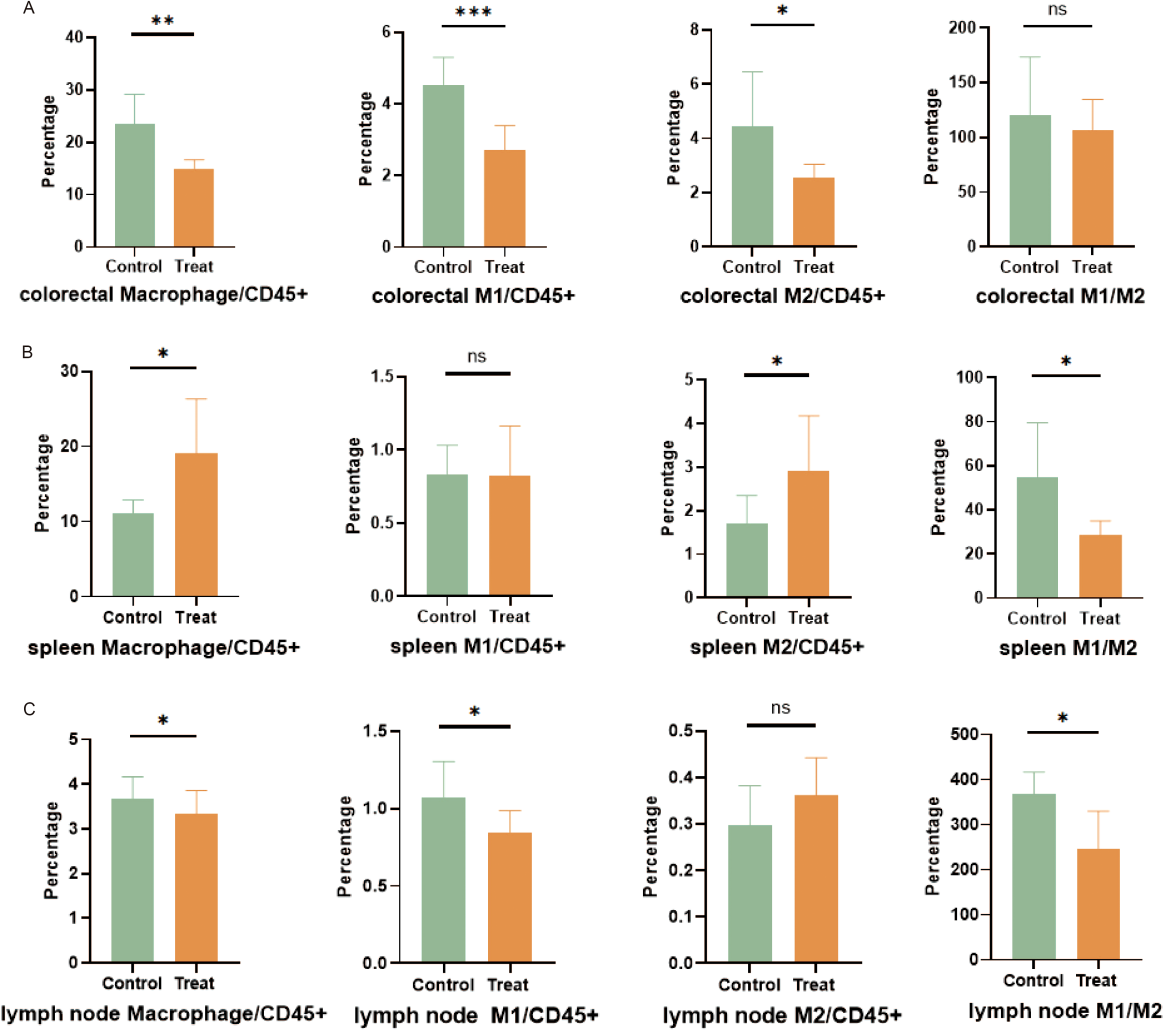
Flow cytometry was used to analyze cell subsets in each tissue of mice (n=8). **(A)** Evaluation and quantification of macrophages in the colorectal. **(B)** Evaluation and quantification of splenic macrophages. **(C)** Evaluation and quantification of macrophages in mesenteric lymph nodes. The results were represented as mean ± SD. *p < 0.05; **p < 0.01; ***p < 0.001; ns, no significance.

Compared with the control group injected with DMSO, the overall proportion of macrophages in the colorectal tissue of mice in the FX1 treat group was significantly reduced (**Figure 5A**). Additionally, both M1 and M2 proportions were significantly reduced, with the difference in M1 levels between the treatment group and the control group being more pronounced. In contrast to the significant findings regarding macrophage clustering, there was no notable difference in the proportion of stromal cells in the gut between the two groups of mice (data not shown). It is therefore speculated that FX1 does not mitigate intestinal inflammation by directly protecting stromal cells.

The results regarding macrophage clustering differed in spleen and mesenteric lymph nodes. In the spleen of the FX1 treat group, there was a significant increase in macrophage proportion as well as a higher proportion of M2. Therefore, the ratio of M1/M2 was decreased in the Bcl6 inhibitor group (**Figure 5B**). Similarly, while there was a slightly lower overall proportion of macrophages in mesenteric lymph nodes within the FX1 Treat group compared to controls, it was noted that the reducing proportion of M1 leads to the decrease of M1/M2 (**Figure 5C**).

Our data suggests that although FX1 alters relative proportions among macrophage subsets, it reduces differentiation towards pro-inflammatory M1 while favoring anti-inflammatory transformation towards M2. However, further investigation is needed to determine if this has any effect on macrophage activation or their pro-inflammatory state as part of an organism’s physiology.

In conclusion, our findings indicate that FXI has inhibitory effects on chemotaxis and infiltration by macrophages within acute colitis-afflicted mice.

### 3.5 Bcl6 inhibitor directly impacts the secretion of pro-inflammatory cytokines in murine macrophages.

Based on the previous analysis results of flow cytometry, it is evident that FX1 has an impact on the clustering of macrophages. In comparison to the control group, the FX1 treatment group exhibited a reduction in the proportion of M1. However, it remains unclear whether FX1 has any effect on the proinflammatory function of M1. Therefore, we conducted *in vitro* cell experiments using RAW264.7 and Caco2 cells.

**Figure 6:**
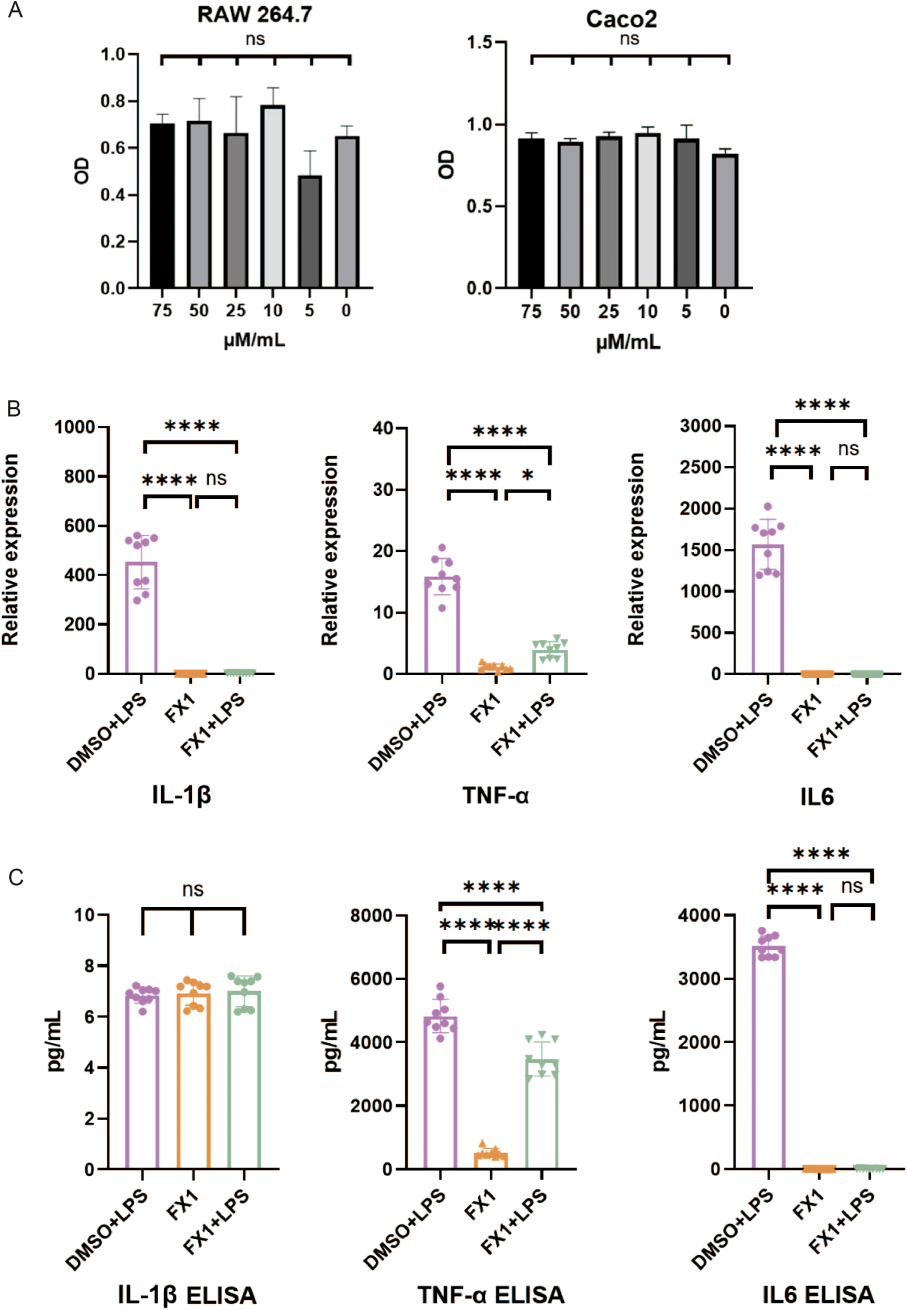
Effects of FX1 on secretion of inflammatory cytokines in macrophages *in vitro*. **(A)** RAW264.7 cells/Caco2 cells were incubated with different concentrations of FX1 for 4 hours. CCK-8 assay was used to confirm cell viability.(n=3). **(B)** RAW264.7 cells were incubated with FX1 for 4 hours and then exposed to LPS for 24 hours. And mRNA levels of IL-1β, TNFα and IL6 in RAW264.7 cells were measured by RT-qPCR (n=9). **(C)** RAW264.7 cells were incubated with FX1 for 4 hours and then exposed to LPS for 24 hours. IL-1β, TNFα and IL6 levels of RAW264.7 cell supernatant were measured by ELISA (n=9). The results were represented as mean ± SD. *p < 0.05; ****p < 0.0001; ns, no significance.

In order to avoid using FX1 concentrations that may have toxic side effects on cell growth, we initially assessed the effect of FX1 on RAW 264.7 cell survival. Cells were exposed to various concentrations of FX1 (ranging from 0μM/mL to 75 μM/mL) for 4 hours, and subsequently their viability was evaluated using a CCK-8 assay. The results indicated no significant difference in cell viability when RAW 264.7 and Caco2 cells were treated with FX1 at concentrations ranging from 0 to 75 μM/mL (**Figure 6A**). After conducting a comprehensive review of existing literature, we selected a concentration of 50 μM/mL for further experiments (15, 25).

To investigate the impact of FX1 on macrophage activation, RAW 264.7 cells were exposed to 50μM/mL of FX1 for 4 hours followed by LPS (at a concentration of 1μg/ml) for an additional period of time lasting up to 24 hours. The supernatant was collected for ELISA, and the cells were frozen in TRIzol at -80°C for RT-qPCR. The findings revealed that FX1 significantly decreased mRNA expression levels TNFα, IL-1β and IL6 in LPS-activated RAW264.7cells (**Figure 6B**). Additionally, FX1 down-regulated the secretion of TNFα and IL6 proteins. However, no significant changes were observed in IL-1β protein levels (**Figure 6C**). These findings suggest that FX1 significantly inhibits production of pro-inflammatory cytokines in macrophages.

### 3.6 Bcl6 inhibitor indirectly protects the intestinal epithelial barrier by inhibiting the inflammatory response of macrophages

**Figure 7:**
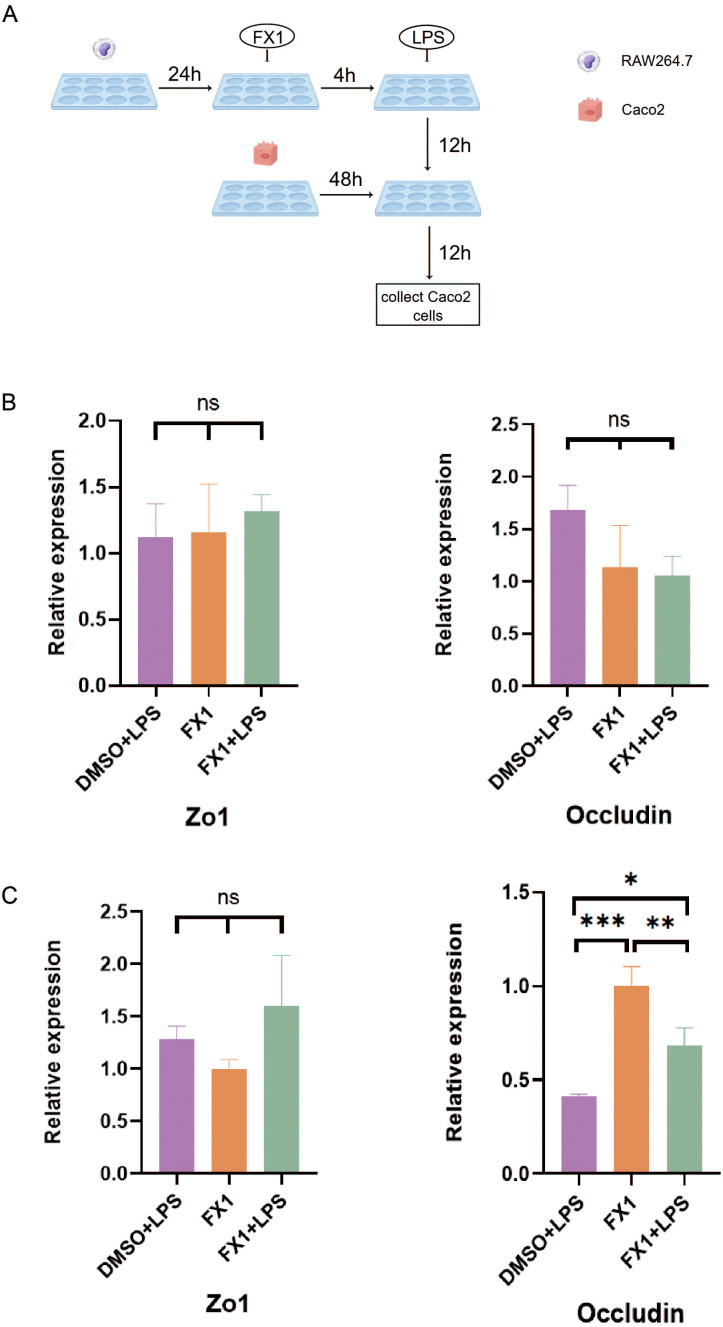
Effect of FX1 on intestinal barrier simulated by Caco2 cells. **(A)** Flow chart of co-culture operation (By Figdraw). **(B)** Caco2 cells were incubated with 50µM/mL FX1 for 4 hours and then stimulated by LPS. The expression of tight junction proteins were detected (n=3). **(C)** Expression of tight junction protein mRNA in Caco2 cells after incubation with RAW264.7 cells supernatant for 12 hours (n=3). The results were represented as mean ± SD. *p < 0.05; **p < 0.01; ***p < 0.001; ns, no significance.

To investigate the protective effect of FX1 on the intestinal epithelial barrier *in vitro*, we simulated the physiological environment of the intestine and directly treated Caco2 cells with FX1 before LPS stimulation. The results showed that FX1 treatment had no significant effect on the mRNA expression of tight junction proteins Zo1 and Occludin in Caco2 cells after LPS stimulation (**Figure 7B**). Co-culture experiments were then performed to determine whether FX1 protection of the intestinal epithelial barrier was mediated by inhibition of macrophage inflammation. RAW 264.7 cells in both the experimental and control groups were treated with FX1 or DMSO for 4 hours, followed by stimulation with LPS for 24 hours. Subsequently, the supernatant was collected. The collected supernatant from RAW 264.7 cells was then incubated with adherent Caco2 cells for 12 hours, after which RNA was extracted from the Caco2 cells for RT-qPCR analysis. (**Figure 7A**). The results showed that the expression of Occludin was significantly increased in Caco2 cells cultured with RAW246.7 macrophage supernatant treated by FX1 and FX1+LPS (**Figure 7C**). Although there was no significant difference in the expression of Zo1, the expression trend was higher after FX1 treatment than without the treatment(**Figure 7C**). These combined results suggest that FX1 may indirectly maintain an appropriate epithelial barrier by inhibiting macrophage secretion of pro-inflammatory mediators, rather than directly protecting intestinal epithelial cells.

## 4 Discussion

This study investigated the effects of the BCL6 inhibitor FX1 on DSS-induced colitis in mice. Throughout the course of the disease, treatment with FX1 significantly alleviated intestinal inflammation, reduced the proportions of macrophages and CD4+ T cells within the intestinal environment, and preserved the integrity of the intestinal epithelial mucosa. Further investigations revealed that FX1 decreased pro-inflammatory factor secretion by macrophages while enhancing tight junction protein expression levels. Our findings demonstrate the efficacy of BCL6 inhibitor FX1 in treating colitis in mice. In recent years, it has been reported that the down-regulation of Bcl6 can promote colonic epithelial cells apoptosis and M1 macrophage polarization while exacerbating the progression of ulcerative colitis (UC) (26). Additionally, silencing Bcl6 may lead to unexpected outcomes such as atherosclerosis and xanthomatous tendinitis (27). However, our animal studies have indicated that Bcl6 possesses potential therapeutic effects on colitis. Notably, the injection of FX1, a specific inhibitor of Bcl6, did not worsen DSS-induced intestinal inflammation in mice. Furthermore, targeted inhibition of Bcl6 significantly alleviated colitis symptoms in these animals, as assessed by Disease Activity Index (DAI) scores and observed changes in the colon.

Bcl6 functions as a transcriptional suppressor, influencing both macrophages and T cell subsets (11, 28). This study utilized *in vivo* experiments to demonstrate that the application of Bcl6 inhibitors could mitigate colon inflammation induced by DSS in mice. Notably, the expression levels of intestinal inflammatory factors significantly decreased following treatment. Further investigations revealed an overall reduction in macrophage populations within the gut and other immune tissues. *In vitro* assays indicated that Bcl6 inhibitors possess the capacity to diminish cytokine production from macrophages and inhibit pro-inflammatory effects. In macrophages, Bcl6 is recognized as a critical regulator of IL-1β and IL18. It promotes Th2 cell differentiation in allergic diseases while inhibiting chemokine Ccl2 expression and NF-κB activity, thereby mediating negative regulation of the innate immune response (29). Additionally, Bcl6 directly inhibits the targeting of chemokines and pro-inflammatory cytokines by specifically binding to target gene promoters within macrophages (17, 30). These findings suggest that Bcl6 plays a role in suppressing acute inflammatory responses (31, 32). However, it has been observed that Bcl6 ^-/-^ mice exhibit a high incidence of severe TH2 inflammatory disease (31), whereas Bcl6^BTBMUT^ mice do not present with inflammation (33). Consequently, some researchers propose that the inhibitory effect of Bcl6 on macrophage inflammation is independent of BTB domain regulatory factors; thus, employing FX1 will not exacerbate the inflammatory response (33). Nevertheless, this perspective does not fully elucidate the anti-inflammatory properties associated with Bcl6 inhibitors.

In another mouse sepsis study, FX1 was shown to inhibit the phosphorylation of JNK, p38, and ERK in LPS-activated RAW 264.7 cells. Additionally, it inhibited the NF-κB signaling pathway to suppress LPS-induced pro-inflammatory cytokines. CHIP experiments demonstrated that following FX1 treatment, there was enhanced binding of Bcl6 to the promoters of IL6, CXCR4, and MCP-1 (17). This indicates that FX1 may block interactions between Bcl6 and its co-inhibitors, thereby enhancing Bcl6’s binding to target gene promoters and leading to the inhibition of chemokines and pro-inflammatory cytokines.

Interestingly, while the Bcl6 inhibitor FX1 inhibits various cytokines, it does not affect TNFα (17). Our results demonstrated that the BCL6 inhibitor FX1 significantly inhibited inflammatory factors such as IL6 and IL-1β in a DSS-induced colitis model, while showing no significant effect on TNFα. The potential mechanism underlying this selective regulatory phenomenon may be attributed to the specificity of BCL6 target genes and the compensatory activation of its signaling pathways. Previous studies have indicated that Bcl6 functions as a transcriptional suppressor by binding to the promoter regions of STAT3-dependent inflammatory factors (such as IL6 and IL23) and recruiting HDAC complexes to inhibit their transcription (34). In contrast, the transcriptional regulation of TNFα is primarily governed by the NF-κB pathway (35). In the DSS colitis model, TLR4/MyD88 signaling consistently activates the IKK-NF-κB axis, thereby driving TNFα expression (36, 37), potentially bypassing any inhibitory effects exerted by Bcl6. This hierarchical regulatory network suggests that FX1 inhibits certain inflammatory factors through targeting the STAT3-BCL6 axis. However, the escape of TNFα may reflect the complexity inherent in multi-pathway interactions within an inflammatory microenvironment. This suggests that FX1 targets other inflammatory pathways and may be particularly beneficial in cases where anti-TNFα therapy is ineffective.

Our experimental results demonstrated that Bcl6 inhibitors significantly reduced the damage to intestinal epithelial cells and the secretion of inflammatory factors in colitis mice. Previous studies have indicated that macrophages played a crucial role in maintaining intestinal immune homeostasis and lamina propria monocytes and M1 macrophages infiltrating intestinal tissue directly compromise the epithelial barrier by dysregulating tight junction proteins and inducing apoptosis in epithelial cells, thereby exacerbating intestinal inflammation associated with IBD (38). We observed similar findings through IHC and flow cytometry analyses conducted on mouse colorectal tissues. Additionally, we noted a decrease in the M1/M2 phenotype across all tissues within the Bcl6 inhibitor group. The imbalance of this phenotype has been linked to worsening colitis in IBD mouse models (39, 40). Bcl6 inhibitors also modulate the detrimental effects of macrophages on intestinal epithelial cells. Marianne et al. reported that non-polarized macrophages and selectively activated M2 macrophages enhance the integrity of the intestinal epithelial barrier, whereas monocytes and pro-inflammatory M1 macrophages increase barrier permeability (41). Our cellular experiments further corroborated these findings: after inhibiting inflammatory factor secretion from macrophages, we observed an increase in tight junction protein expression within Caco2 cells. We noticed that Occludin has significant difference on protein levels, while there were no statistical differences mRNA level. The RT-qPCR data exhibited substantial intra-group variability (as reflected in error bar magnitude) combined with limited sample size (n=5). These constraints likely reduced statistical power below the threshold required to detect significant differences. In addition, It has also been documented that post-transcriptional regulation or altered protein stability could amplify protein-level changes without proportional mRNA shifts (42). Similar results were found for measurements of IL-1β. Maturation and secretion of IL-1β in mononuclear cells and macrophages require multi-step activation. LPS alone may only induce the expression of inactive IL-1β (pro-IL-1β), but the maturation and secretion of IL-1β are controlled by inflammasome (43, 44). Several studies have shown that the secretion of IL-1β requires inflammasome activation (45, 46), while TNFα and IL6 are directly induced by the MyD88/TRIF pathway after TLR4 activation (35). Therefore, we consider that the inconsistencies of IL-1β in mRNA and protein levels are mainly attributed to its unique post-translational processing mechanism, and LPS alone is insufficient to activate the inflammasome, resulting in the inability of pro-IL-1β to mature secretion (47). In addition, limitations of detection methods, such as some ELISA kits only detect mature IL-1β (17 kDa), while cells may accumulate uncut pro-IL-1β (31 kDa) or secrete insufficient amounts, resulting in false negatives. IL-1β secretion may be lower than ELISA detection limit.

In addition, our study also analyzed T cell subsets, and we observed that the proportion of CD4^+^ T cells in the spleen and colorectal of mice in the treatment group was reduced, but there was no significant difference in the intestinal Treg subsets (**Supplementary Figure S2**). Similar animal efficacy experiments showed that the BCL6 inhibitor extended the survival of chronically rejected heart grafts and also reduced the proportion and number of spleen CD4+T cells, effector CD4+T cells, proliferating CD4+T cells, and Tfh cells in chronically rejected mice (14). It was previously reported that down-regulating BCL6 expression in DSS-induced mice can affect Tfh and Tfr cell contents as well as the ratio of Tfh/Tfr cells, leading to an improvement in intestinal inflammatory response (48). Additionally, recent studies have shown that ICOS can increase BCL6 expression through molecular chaperone-mediated autophagy (CMA), resulting in excessive Tfh cell response and GC response (49). This suggests that Bcl6 can influence CD4+T cell differentiation and promote inflammation. However, the Bcl6 inhibitor can reduce both proportion and number of CD4+T cells during inflammation processes; thus it may play a role in alleviating intestinal inflammation.

In summary, this study demonstrated both *in vivo* and *in vitro* that the BCL6 inhibitor can alleviate acute colitis induced by DSS in mice. Treatment with the BCL6 inhibitor significantly reduces the migration of macrophages to the colon and rectum, as well as diminishes the secretion of inflammatory factors from these macrophages. These findings underscore the potential of BCL6 as a therapeutic target for IBD and highlight the promising role of BCL6 inhibitors in its treatment. Furthermore, no individual or cytotoxic effects associated with FX1 were observed in this study, alleviating concerns regarding the risks linked to its application.

## Data Availability

The original contributions presented in the study are included in the article/**Supplementary Material**. Further inquiries can be directed to the corresponding authors.
