# Supplementary figures and images for: Inhibitior of Bcl6 by FX1 protects DSS induced colitis mice through anti-inflammatory effects
Source: Front Immunol. 2025 May 9;16:1558845. doi: 10.3389/fimmu.2025.1558845 (PMC12098098; doi:10.3389/fimmu.2025.1558845)

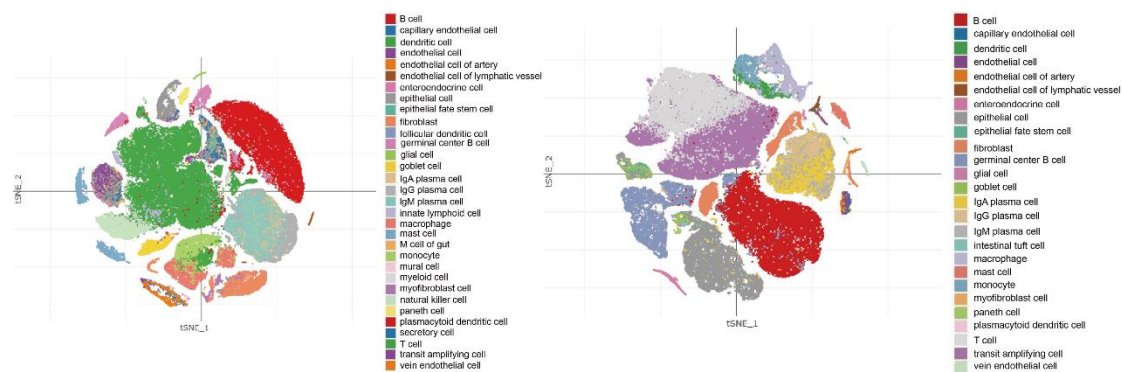

**Supplementary Figure1:** Single-cell tSNE in IBD and non-IBD patients.

Supplement: Supplementary file 2 [file Image1.pdf]

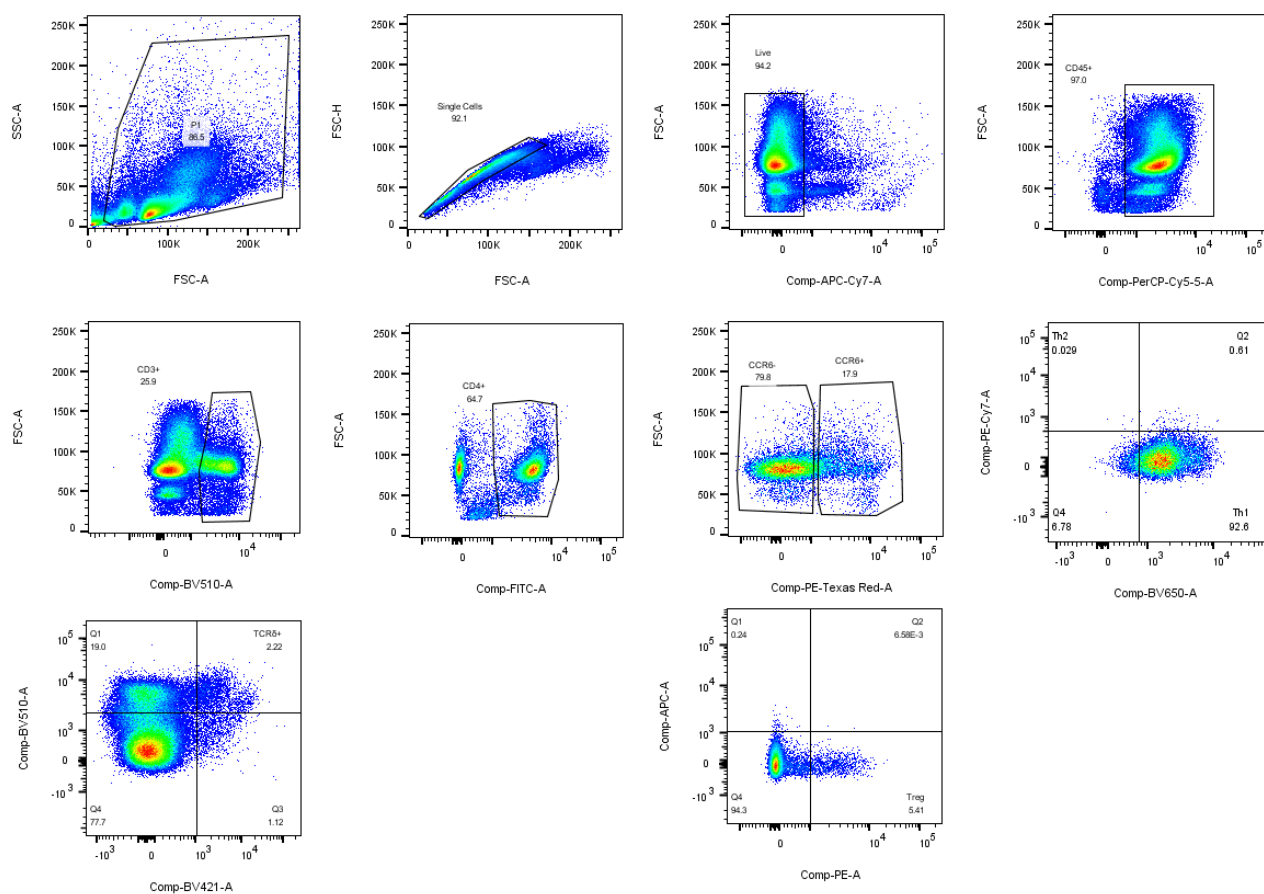

**Supplementary Figure 2: T Cell Subpopulation Gating Strategy.**

Supplement: Supplementary file 3 [file Image2.pdf]

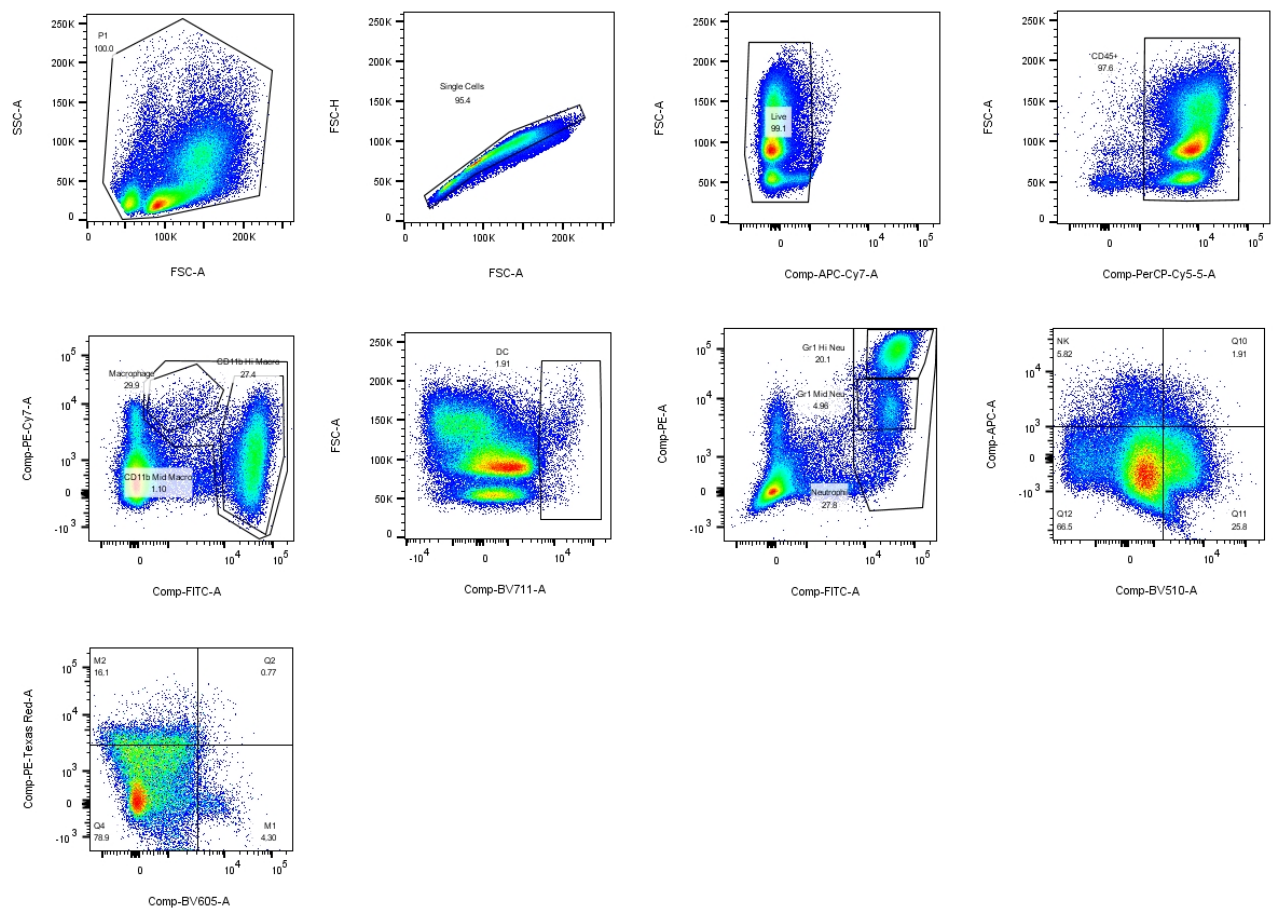

**Supplementary Figure 3: Myeloid Cell Gating Strategy.**

Supplement: Supplementary file 4 [file Image3.pdf]
