# Supplementary material for: Inhibitior of Bcl6 by FX1 protects DSS induced colitis mice through anti-inflammatory effects
Source: Front Immunol. 2025 May 9;16:1558845. doi: 10.3389/fimmu.2025.1558845 (PMC12098098; doi:10.3389/fimmu.2025.1558845)
Supplement: Supplementary file 5 [file Image4.pdf]

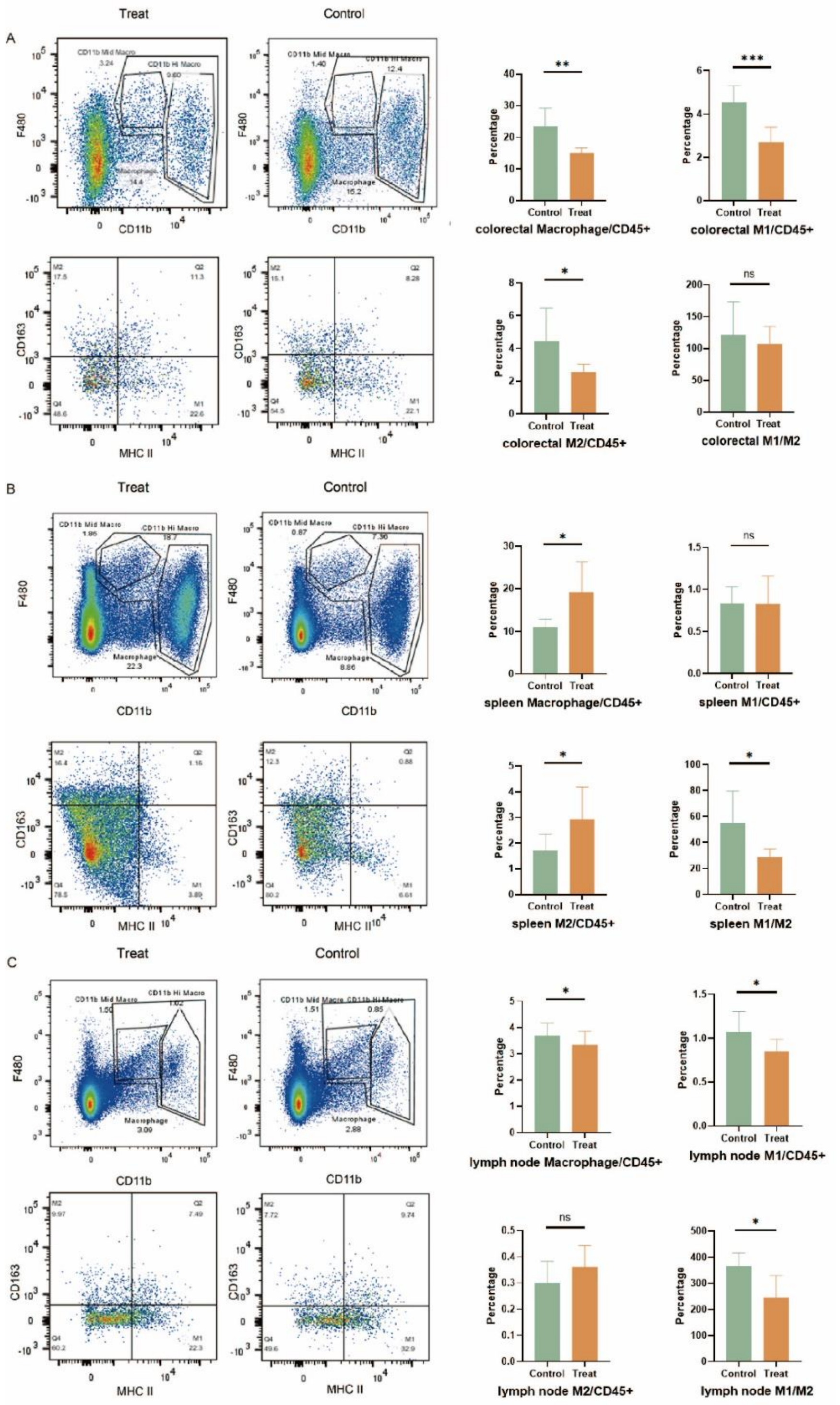

Supplementary Figure 4: Flow cytometry scatter plots and bar plots of macrophages in various tissues. (A) Flow scatter diagram of colonic macrophages. (B) Flow scatter diagram of splenic macrophages. (C) Flow scatter diagram of mesenteric lymph node macrophages.
