# Supplementary material for: Inhibitior of Bcl6 by FX1 protects DSS induced colitis mice through anti-inflammatory effects
Source: Front Immunol. 2025 May 9;16:1558845. doi: 10.3389/fimmu.2025.1558845 (PMC12098098; doi:10.3389/fimmu.2025.1558845)
Supplement: Supplementary file 6 [file Image5.pdf]

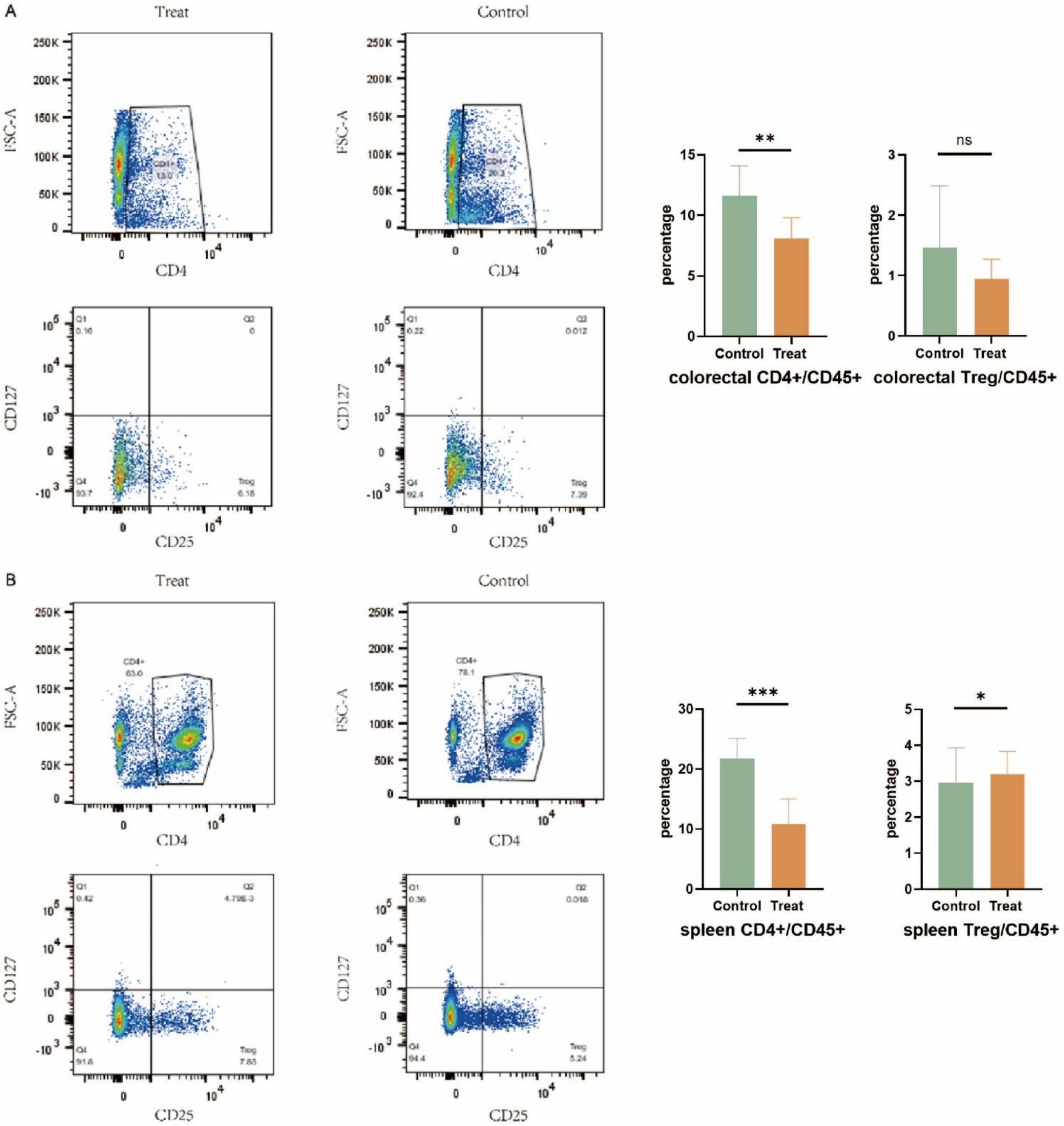

Supplementary Figure 5: Flow cytometry scatter plot and histogram of $CD4^{+}$T cells and Treg cells in each tissue. (A) Flow scatter plots of $CD4^{+}$ T cells and Treg cells in the colon. (B) Flow scatter plots of $CD4^{+}$ T cells and Treg cells in spleen.
