# Supplementary material for: Inhibitior of Bcl6 by FX1 protects DSS induced colitis mice through anti-inflammatory effects
Source: Front Immunol. 2025 May 9;16:1558845. doi: 10.3389/fimmu.2025.1558845 (PMC12098098; doi:10.3389/fimmu.2025.1558845)
Supplement: Supplementary file 7 [file Table1.docx]

## Supplementary Table:

**Supplementary** **Table** **1 Calculation of DAI score**^[1]^

| Score | Weight loss | Stool Characters | Hematochezia |
| --- | --- | --- | --- |
| 0 | Normal | Normal (well-formed pellets) | Not observed |
| 1 | 1-5% | Normal | Not observed |
| 2 | 6-10% | Loose (pasty stools that do not stick to the anus) | Occult |
| 3 | 11-15% | Loose (pasty stools that do not stick to the anus) | Occult |
| 4 | > 15% | Diarrhea (Liquid stools that stick to the anus) | Gross bleeding |

[1] Chaudhary, G.; Mahajan, U. B.; Goyal, S. N.; Ojha, S.; Patil, C. R.; Subramanya, S. B. Protective Effect of Lagerstroemia Speciosa against Dextran Sulfate Sodium Induced Ulcerative Colitis in C57BL/6 Mice. *Am J Transl Res* **2017**, *9* (4), 1792–1800.
