# Supplementary material for: Inhibitior of Bcl6 by FX1 protects DSS induced colitis mice through anti-inflammatory effects
Source: Front Immunol. 2025 May 9;16:1558845. doi: 10.3389/fimmu.2025.1558845 (PMC12098098; doi:10.3389/fimmu.2025.1558845)
Supplement: Supplementary file 8 [file Table2.docx]

## Supplementary Table:

**Supplementary** **Table** **2 Histological scoring criteria**^[2]^

| Category | Description | Score |
| --- | --- | --- |
| Extent of inflammation |  |  |
|  | None | 0 |
|  | Mild inflammation | 1 |
|  | Moderate inflammation | 2 |
|  | Severe inflammation | 3 |
| Inflammatory infiltration area |  |  |
|  | None | 0 |
|  | Mucosal layer | 1 |
|  | Submucous laye | 2 |
|  | Muscular layer | 3 |
| Crypt damage |  |  |
|  | None | 0 |
|  | 0- 1/3 crypt deformated or destroyed | 1 |
|  | 1/3-2/3 crypt deformated or destroyed | 2 |
|  | The crypt was almost lost and only the epithelium was retained | 3 |
|  | The crypt and epithelium were completely destroyed | 4 |

[2] Wang Q, Liu Y, Gao L, Zhang L,Wang J. Study on the Protective Effect and Mechanism of Umbilicaria esculenta Polysaccharide in DSS-Induced Mice Colitis and Secondary Liver Injury*.* J Agric Food Chem, 2024. **72**(19): p. 10923-10935.
